# Supplementary material for: Sodium–Glucose Cotransporter 2 Inhibitor Combined with Conventional Diuretics Ameliorate Body Fluid Retention without Excessive Plasma Volume Reduction
Source: Diagnostics (Basel). 2024 Jun 5;14(11):1194. doi: 10.3390/diagnostics14111194 (PMC11171863; doi:10.3390/diagnostics14111194)
Supplement: Supplementary file 1 [file diagnostics-14-01194-s001.zip › diagnostics-2976886-supplementary.pdf]

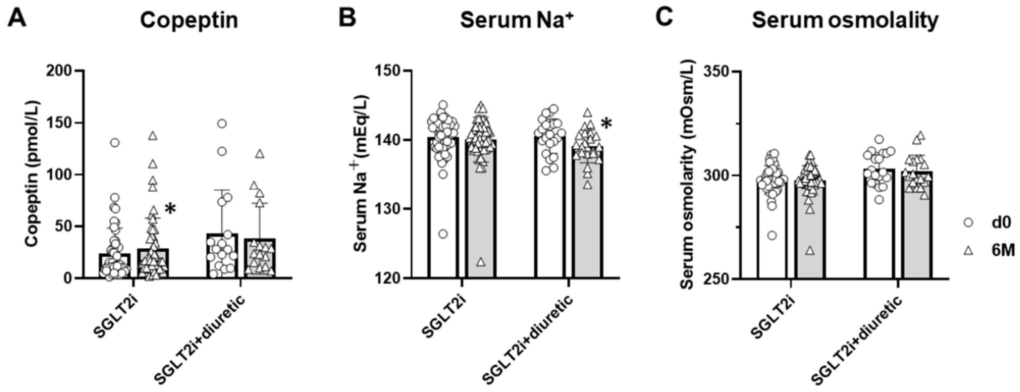

Supplementary Figure S1. Absolute changes in copeptin (A), serum Na^+, and serum osmolality (C) from day 0 (d0) to 6 months (6M). * p<0.05 vs. d0

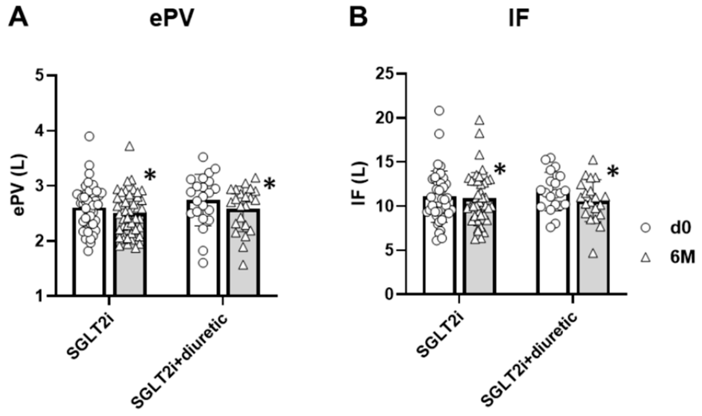

Supplementary Figure S2. Absolute changes in estimated plasma volume (ePV) (A) and interstitial fluid (IF) (B) from day 0 (d0) to 6 months (6M). * p<0.05 vs. d0

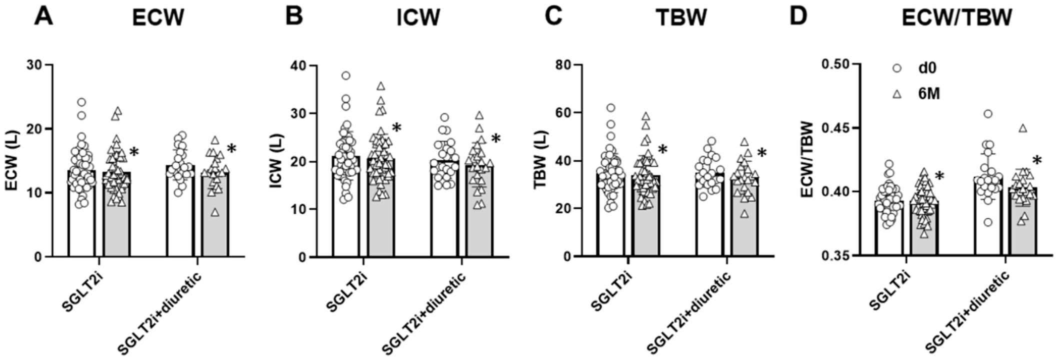

Supplementary Figure S3. Absolute changes in extracellular water (ECW) (A), intracellular water (ICW) (B), total body water (TBW), and ECW/TBW from day 0 (d0) to 6 months (6M). * p<0.05 vs. d0

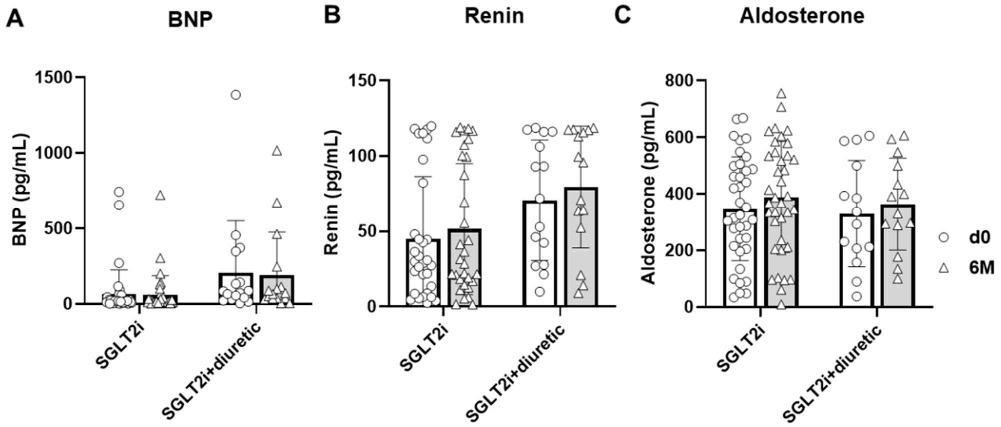

Supplementary Figure S4. Absolute changes in brain natriuretic peptide (BNP) (A), serum renin (B), and serum aldosterone (C) from day 0 (d0) to 6 months (6M).
